# In Vitro and In Vivo Models to Study the Zoonotic Mosquito-Borne Usutu Virus

**DOI:** 10.3390/v12101116

**Published:** 2020-09-30

**Authors:** Emna Benzarti, Mutien Garigliany

**Affiliations:** Fundamental and Applied Research for Animals & Health (FARAH), Faculty of Veterinary Medicine, University of Liège, Sart Tilman B43, B-4000 Liège, Belgium; ebenzarti@uliege.be

**Keywords:** USUV, experimental model, in vitro, in vivo, pathogenesis

## Abstract

Usutu virus (USUV), a mosquito-borne zoonotic flavivirus discovered in South Africa in 1959, has spread to many European countries over the last 20 years. The virus is currently a major concern for animal health due to its expanding host range and the growing number of avian mass mortality events. Although human infections with USUV are often asymptomatic, they are occasionally accompanied by neurological complications reminiscent of those due to West Nile virus (another flavivirus closely related to USUV). Whilst USUV actually appears less threatening than some other emergent arboviruses, the lessons learned from Chikungunya, Dengue, and Zika viruses during the past few years should not be ignored. Further, it would not be surprising if, with time, USUV disperses further eastwards towards Asia and possibly westwards to the Americas, which may result in more pathogenic USUV strains to humans and/or animals. These observations, inviting the scientific community to be more vigilant about the spread and genetic evolution of USUV, have prompted the use of experimental systems to understand USUV pathogenesis and to boost the development of vaccines and antivirals. This review is the first to provide comprehensive coverage of existing in vitro and in vivo models for USUV infection and to discuss their contribution in advancing data concerning this neurotropic virus. We believe that this paper is a helpful tool for scientists to identify gaps in the knowledge about USUV and to design their future experiments to study the virus.

## 1. Introduction

**Figure 1:**
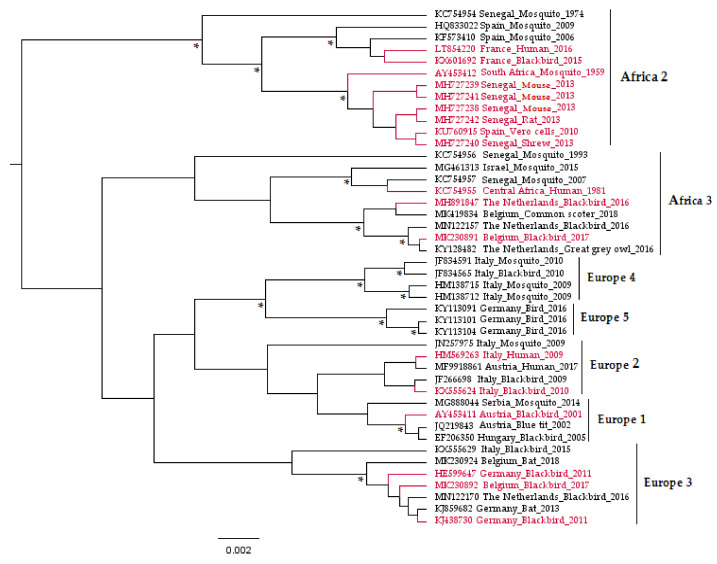
Phylogenetic tree representing the placement of Usutu virus (USUV) strains used to infect experimental models in vitro and in vivo based on partial non-structural 5 genes. The asterisks (*) indicate a support value from Bayesian posterior probabilities >70%. To improve visualization, Africa 1 lineage (KC754958 Central African Republic_Mosquito_1969) is not represented in the figure, and phylogenetic positions of USUV experimentally used in the laboratory are red-colored. Taxon information includes the GenBank accession number, country, and host in which the virus was detected and detection year. The scale bar indicates the mean number of nucleotide substitutions per site.

Usutu virus (USUV) is a zoonotic mosquito-borne flavivirus related to Japanese encephalitis (JEV) and West Nile (WNV) viruses [1]. Responsible for recurrent epizootics in the European avifauna [2,3,4,5,6,7,8], USUV has recently attracted the attention of the scientific community due to its association with potentially severe neurological disorders in humans [9,10,11,12]. It is an enveloped virus of 40–65 nm in size with an icosahedral symmetric nucleocapsid and a monocistronic single-stranded viral RNA (vRNA) genome of positive polarity and approximately 11 kb [1]. This vRNA is composed of a cap at the 5’ end, followed by a short non-coding untranslated region (UTR) in 5′, then a single open-reading-frame that encodes a polyprotein, and finally a 3′ UTR (about 400 to 700 nucleotides) lacking a poly-adenylated sequence [13]. USUV viral proteins are a product of the polyprotein cleavage, during or after translation, by viral and cellular proteases [13]. These proteins comprise three structural proteins (capsid C, Membrane precursor prM, and envelope E), which form the virion; seven nonstructural proteins (NS1, NS2A, NS2B, NS3, NS4A, 2K, NS4B, and NS5) involved in the viral replicative cycle and in complex mechanisms to evade the host immune responses [14,15]; and one peptide 2K, which serves as a signal sequence for the NS4B protein translocation into the endoplasmic reticulum lumen [16]. The genetic variability of USUV has been explored through phylogenetic studies conducted on the complete viral sequences, as well as on genes encoding the envelope (E) and non-structural 5 (NS5) proteins [17]. These analyses grouped the USUV strains into eight distinct lineages: Africa 1, 2, and 3 and Europe 1, 2, 3, 4, and 5 (Figure 1).

USUV was isolated for the first time from a *Culex neavei* mosquito captured near the Usutu river in Ndumu, South Africa, in 1959 [18,19]. Subsequently, USUV circulation in the African continent has been detected in several countries: Senegal, Central African Republic, Nigeria, Uganda, Burkina Faso, Ivory Coast, Tunisia, Morocco, and Algeria [20,21,22,23]. Until 2001, USUV was considered as exclusively African, non-fatal for wild birds or domestic animals, and exceptionally zoonotic [22]. In 2001, USUV was isolated from blackbirds (*Turdus merula*) found dead during an epizootic that affected the resident passerines and *Strigiformes* in Austria [4]. Retrospective analyses have shown that the high mortality of blackbirds in Tuscany (Italy) in 1996 was also attributed to this virus [24]. In the following years, USUV circulation was identified in many countries in western, southern, and central Europe: United Kingdom (2001–2002) [25], Czech Republic (2005) [26], Hungary (2005) [27], Poland (2006) [28], Spain (2006) [29], Switzerland (2006) [30], Serbia (2009–2010) [31], Greece (2010) [32], Germany (2011) [33], Slovakia (2012–2014) [34], Belgium (2012) [35], France (2015) [36], and The Netherlands (2016) [6]. In many of these countries, USUV has managed to establish an endemic mosquito–bird life cycle and to co-circulate with WNV [37].

To date, USUV has been detected in mosquitoes belonging to seven genera (*Aedes*, *Anopheles*, *Coquillettidia*, *Culex*, *Culiseta*, *Mansonia*, and *Ochlerotatus*) [37]. However, it seems to be most often associated with *Culex pipiens* [22,29,38,39,40]. The main natural reservoir hosts of USUV are birds; the virus presence was demonstrated to date in 101 bird species belonging to 18 orders and 38 families [7,8,22,41]. However, the natural virulence spectrum of USUV seems rather limited, with a marked virulence in the European blackbird (*Turdus merula*) [42], house sparrow (*Passer domesticus*) [43], grey owl (*Strix nebulosa*) [44], and common scoter (*Melanitta nigra*) [7]. In these species, prostration, disorientation, locomotor disorders, and death may occur [7,42,43,44]. The two macroscopic lesions most commonly observed at autopsy are splenomegaly and hepatomegaly [2,42]. Pathohistological analysis revealed inflammatory and necrotic lesions, with histiocytic and lymphoplasmacytic infiltrates, have been described in the heart, lung, liver, kidney, spleen, and brain of the infected birds [2,27,42,45]. Although the virus was isolated from mammalian species, namely rodents (*Mastomys natalensis*, *Crocidura spp.*, and *Rattus rattus*) [46] and Chiroptera (*Rousettus aegyptiacus* [47] and *Pipistrellus pipistrellus* [2,48]), no pathological signs could be observed in these hosts and their potential role as a reservoir for this arbovirus is still questionable. Other mammals, such as equids [21,31,34,49,50,51], dog [21], wild boar (*Sus scrofa*) [52], red deer (*Cervus elaphus*) [53], tree squirrel (*Sciurus vulgaris*) [54], Malayan tapir (*Tapirus indicus*), chimpanzee (*Pan troglodytes*), giant panda (*Ailuropoda melanoleuca*), common eland (*Taurotragus oryx*), and white rhinoceros (*Ceratotherium simum*) [55], as well as reptiles (green lizards (*Lacerta viridis*) [56]), presented neutralizing antibodies specific for USUV and may act as incidental hosts.

In humans, USUV infection (like WNV) is usually asymptomatic. More than 80 cases of subclinical infections have been described in blood donors or healthy patients in Italy, Serbia, the Netherlands, and Germany during the surveillance of WNV circulation [57,58,59,60,61,62,63,64]. Clinical disease with moderate flu-like (rash, fever, and headache) manifestations may also occur [65]. The neurotropism of USUV represents a growing concern for human health. In more than 32 cases to date, severe neurological disorders, including facial paralysis, encephalitis, meningitis, and meningoencephalitis, in both immunocompromised and immunocompetent patients have been observed [12,66]. These severe acute human cases, along with the avian mass mortality induced by this virus in Europe and numerous similarities with WNV biology and clinical manifestations, have prompted the development of experimental models to clarify the mechanisms underlying USUV pathogenesis and transmission. Besides, given that no approved effective therapeutics and no licensed vaccines against USUV exist so far for humans or birds, some of these models were used for their development. This is the first review to focus on in vitro and in vivo models of infection with USUV and summarize their contribution to clarify USUV pathogenesis and potential countermeasures.

## 2. In Vitro Models

So far, USUV infection has been studied in vitro using two-dimensional (2-D) cell culture systems (cell monolayers). They consist of two cellular systems: human and animal primary cells, which are isolated directly from an animal or human tissues, and immortalized cell lines, established to proliferate indefinitely [67]. Cell cultures are not only useful in virus isolation and serological assays but also to characterize virus tropism and pathogenesis, and to validate drug and vaccine candidates. However, in vitro methods are artificial and present many limitations [68]. Besides, although considered to be genetically and phenotypically homogenous, the same cell line from different laboratories might display biological differences [69]. Thus, researchers should interpret and discuss the results driven from these systems with caution.

To date, around 20 articles (Table 1 and Table 2) have studied USUV growth kinetics using these in vitro systems. This number is very small compared with that of publications dealing with other mosquito-borne flaviviruses, such as WNV (around 110) or with Dengue virus (over 200) [70], which explains, at least in part, the scarcity of information about USUV infection and control. The USUV prototype strains SAAR-1776 (GenBank: AY453412, Culex neavei), which were isolated by intracerebral inoculation of newborn mice [18], and Vienna 2001 (GenBank: AY453411, Blackbird) were mainly used in these experiments. Nevertheless, other strains representative of different lineages have been investigated for their biology (Figure 1). No strains from Europe 4 and Europe 5 lineages have been tested yet for their pathogenicity either in vitro or in vivo. Further, only one study [71] compared the replication kinetics, indicative of the virulence of four USUV strains (Africa 3 and Europe 1, 2, and 3 lineages), in chicken embryo primary cells and found marked replication of the Europe 3 strain compared to the others. However, in vivo studies using chicken embryos revealed similar pathogenicity between these strains, indicating the importance of combining different models from which to draw robust conclusions. Nevertheless, in vitro studies are further warranted, particularly to unravel a potential difference in the pathogenicity of USUV strains in humans. 

### 2.1. USUV Cellular Tropism

To date, the virus has been shown to infect a large spectrum of cells from 23 mammalian species, two avian species, and one reptile (turtle, *Terrapene carolina*) (Table 1 and Table 2). The first USUV in vitro replication assay was performed in porcine kidney (PK) cells in 1969 [72]. Later, Bakonyi et al. (2005) demonstrated USUV replication in a wide range of cells. However, only African green monkey kidney cells (Vero), PK-15 pig epithelial cells, and goose embryo fibroblasts have developed cytopathic effects (CPE) [73]. Like other flaviviruses, USUV replicates efficiently in Vero and mosquito (*Aedes albopictus*) C6/36 cells, which are commonly used for virus isolation from both clinical and animal (birds/rodents/mosquito) samples [44,46] and often after replication in these cells, other cellular or animal models are used. The particular susceptibility and the extent of CPE observed in Vero cells explain their use for virus culture and viral titer studies, such as 50% tissue culture infectious dose, TCID_50_, and plaque reduction neutralization tests [5]. In these cells lacking the interferon (IFN)-α and IFN-β genes [74], USUV infection activates cellular stress and autophagy, promoting viral replication [75]. Further, USUV can establish a persistent infection for at least 80 days and present full-length and defective viral genomes (DVGs), containing truncations at the 5′ end, which may be a key determinant in the survival and persistence of the infection [76].

Multiple cellular systems were used primarily to investigate USUV tropism. Mammalian cells were further used to explore USUV infection neuropathogenesis, the cell-intrinsic immune response, and/or the effect of antivirals on USUV replication.

USUV shows different replication characteristics in rodent species and rodent-derived cell types. The woodchuck (*Marmota monax)* liver cells (WCH-17, ATCC No: CRL-2082), rat (*Rattus norvegicus*) brain cell line (C6), and hamster (*Mesocricetus auratus)* kidney cell line (BHK-21) were susceptible to USUV infection but did not display CPE [73,77]. However, primary astrocytes, microglial cells, and neurons of a wild-type mouse (*Mus musculus*) supported efficient USUV replication and showed CPE [82]. While a bank vole (*Myodes glareolus*) kidney cell line (BVK168, RRID: CVCL_A014) showed CEPs following USUV infection [81,84], the virus did not replicate at all in the lung cells of this animal and did not show CPE in kidney or brain cells of the common vole (*Microtus arvalis*) [84]. Likewise, USUV could infect human cells from different origins, including the upper respiratory tract, brain, and retina, but only a few of these cells exhibited CPE (Table 2).

The cellular receptors responsible for USUV adherence and internalization into the cell are still largely unknown. In one study addressing this question using human astrocytes, USUV replication was not modulated by blocking either the TAM receptor AXL or the C-type lectin receptor Dendritic Cell-Specific Intercellular adhesion molecule-3-Grabbing Non-integrin (DC-SIGN), indicating that, in contrast to Zika virus (ZIKV), USUV does not use these specific cellular receptors for viral entry [82]. As described for other flaviviruses, the DIII domain of the E protein is the likely receptor-binding domain and the major determinant of virus cellular tropism [90]. In Vero cells persistently infected with USUV, all the truncated genomes were predicted to encode a polyprotein lacking the E protein, most of the NS1, and partially the PrM/M, which suggests that these proteins play a major role in the formation of CPE by triggering cell death due to USUV [76].

### 2.2. USUV Neuropathogenesis

The mechanisms of USUV neuroinvasion and neurovirulence in vitro were explored through infection of astrocytes, neuronal precursors (neural stem cells, NSCs) [82,88], and human brain-like endothelial cells (hBLECs) [85]. Comparatively, WNV (Lineage 1, ITA09 Human from Italy GenBank GU011992.2 and Lineage 2, AUT/2008 goshawk from Austria, GenBank KF179640) could infect and replicate in NSCs with significantly higher efficiency than USUV and both WNV and USUV could replicate more efficiently than ZIKV (strain H/PF/2013, GenBank KJ776791) [88]. In the same study, WNV induced significantly higher levels of IFN type I and inhibitory activity on the IFN response pathway than USUV and ZIKV [88].

The human NSCS (hNSCs) were highly permissive for USUV infection and underwent caspase-3-dependent apoptosis at a higher rate than that following ZIKV infection [82,88]. In addition to direct damage, USUV was suggested to disseminate to neurons via astrocytes, which stop proliferation following USUV infection [82]. USUV infectious particles were efficiently released by hBLECs and were suggested to reach the CNS via this route, without compromising the blood–brain barrier (BBB) integrity [85]. Importantly, in all three models, cytokines (such as CXCL10, IL-1ß, and/or tumor necrosis factor (TNFα)) were upregulated [82,85,88], potentially recruiting inflammatory cells in vivo. These proinflammatory cytokines can induce neuron apoptosis or direct damage in neuronal cells [91] and constitute a double-edged sword in USUV neuropathogenesis, as they participate in viral clearance from the brain but enhance cellular death and cytotoxicity when inflammation is exacerbated [92]. Astrocytes, which constitute the periparenchymal layer of the BBB, were shown to regulate hNSCs replication within the CNS [93] and to increase the permeability of the BBB by producing chemokines, such as IL-6 and TNF [94], and thus, co-culturing these cells with brain endothelial cells might be expected to improve identification of the underlying determinants of CNS infection by USUV in humans.

### 2.3. Cell-Intrinsic Immune Response to USUV Infection

The innate response is the first defense mechanism against the invasion of a pathogen. It is initiated in the skin after the inoculation of an arthropod-borne flavivirus, where various target cells are present, including the dendritic cells (DCs), NK (natural killer) cells, neutrophils, keratinocytes, and fibroblasts [95]. To date, only human DCs have been studied for USUV proinflammatory and antiviral response, and more cellular models are needed, including primary human epidermal keratinocytes or skin explants, for instance, as for ZIKV [14], to characterize the USUV early phase of infection.

Cytokines are fundamental for the coordination of different elements of the immune response. In particular, the IFNs have been studied in a variety of mammalian cell types infected with USUV. In cells of epithelial origin, such as Hep-2, Vero, and primary human nasal epithelial cells, USUV infection triggered type III IFNs [86,89]. However, in Hep-2 and Vero cell lines, the potential of type III IFN to inhibit USUV replication was lower than that of type I IFNs, in contrast to influenza A/H1N1 and WNV [86]. Infection of human astrocytes, primary human nasal epithelial cells, porcine and human monocyte-derived DCs, Vero cells, Hep-2 human cells, and human retina pigmented epithelium (RPE) induced a high level of type I IFN production [82,83,86,87]. USUV was demonstrated to be very sensitive to the antiviral effect of IFNs in human lung epithelial cells A549: The replication was 10-fold lower than that of WNV in the presence of a large variety of subtypes of IFN-α, -β, and γ [87]. The USUV-infected human DCs induced higher levels of type I IFN than those infected with WNV (Lineage 1 strain NY99, GenBank AF196835, and lineage 2 goshawk Austria 361/10, GenBank HM015884) (10–100 fold, depending on the multiplicity of infection) [87]. These findings from both cellular models suggest that USUV is less efficient at inhibiting IFN production than WNV [87]. In Hep-2 and Vero cells, USUV was highly sensitive to the antiviral actions of type I and III IFNs when cells were treated with these cytokines before the viral infection [86]. However, USUV infection weakly induced the production of these types of IFNs on untreated Hep-2 cells [86]. In all the previously mentioned models, the IFN induction did not completely prevent USUV replication, showing that USUV can overcome the type I IFN response and establish a productive infection using an unidentified mechanism [82,86,87]. On the other hand, the lower pathogenicity of USUV to humans compared with that of certain strains of WNV may be due to its greater susceptibility to the host innate response [82,87,88].

### 2.4. Antiviral Assays

Very few antiviral assays have been conducted to date in vitro and none have used human cells and/or brain-derived cell culture systems. Likewise, despite USUV marked pathogenicity for birds, no antivirals were tested in avian cells (or birds in vivo) thus far and only a few trials have been conducted, all in Vero cells [75,76,78,79,80].

The interaction between autophagy and USUV was used as a therapeutic target. Indeed, autophagy inhibitors, such as 3-methyladenine and wortmannin, significantly reduced the USUV replication in Vero cells (3–5 fold) [75]. The host lipid biosynthetic pathways, required for the production of infectious viral particles, were also targeted: The inhibition of the acetyl-CoA carboxylase enzyme by the drugs 5-(Tetradecyloxy)-2-furoic acid (TOFA) and 3,3,14,14-tetramethylhexadecanedioic acid (MEDICA 16) strongly inhibited both WNV and USUV replication in Vero cells [80].

The antiviral strategy of lethal mutagenesis, which uses nucleoside drugs inducing increased virus mutation rates, was investigated with USUV infection in vitro and showed variable efficiency. Favipiravir, a purine analog, was able to inhibit USUV replication only when added to the infected cells during the first 6 h of infection of Vero E6 cells [79]. This molecule, along with another purine analog (ribavirin) and 5-fluorouracil (a pyrimidine analog), led to sustained decreases in virus titers but not to complete viral extinction in Vero cell supernatant media. In the same study, ZIKV was inhibited more efficiently by ribavirin and favipiravir, while USUV replication was affected to a greater extent by 5-fluorouracil [78]. Similarly, a 10-day exposure to favipiravir, ribavirin, or a combination of both drugs could lead to the complete extinction of infectivity and vRNA in the cell-culture supernatant media but not inside Vero cells persistently infected with USUV. Besides, withdrawal after treatment resulted in the recovery of USUV vRNA compatible with the persistence of infectious virus intracellularly [76].

## 3. In Vivo Models

### 3.1. Mosquito Infection Models

Before USUV emergence in Europe, only one study [96] registered experimental infections with USUV in mosquitoes. It showed the susceptibility of *Cx. neavei* to USUV, but no effective transmission to hamsters could be demonstrated [96]. After USUV detection in dead birds and several ornithophilic mosquito species in many European countries, the vector competence of European, African, and even American mosquito populations was addressed through experimental infections of these invertebrate hosts (Table 3 and Table 4). *Cx pipiens* has been used as the major experimental model (in 4/7 studies). This can be justified by the abundance of this vector and the fact that USUV has been frequently detected [97] and co-circulating with WNV [98,99] in biotypes of this mosquito complex collected in nature. Some North American and European populations of *Cx. pipiens pipiens*, *Cx. pipiens molestus*, *Cx. quinquefasciatus*, and/or hybrid forms have shown that both European and African strains of USUV effectively infect their bodies and accumulate in their saliva under laboratory conditions [100,101,102]. However, two UK strains of *Cx. pipiens* infected with a USUV strain of African origin showed a very low vector competence, which could be due to the genetic variability of USUV strains or mosquito populations from the same species [103]. Further, the infectivity of USUV in *Cx. pipiens* showed a pronounced temperature dependency [101]. A clear relationship between the virus titer in the blood sample and the infection rate of *Cx. naevi* was demonstrated [104]. Thus, a range of factors should be carefully considered to compare the competence of a particular mosquito species for the same virus.

The vector competence of *Cx pipiens* for USUV was compared with that for WNV and ZIKV. While none of the tested mosquitoes accumulated ZIKV in the saliva and were considered as incompetent vectors for ZIKV, *Cx. pipiens molestus* and *Cx. pipiens pipiens* were shown to be susceptible to USUV infection and to disseminate the virus in their salivary glands [100]. The infection and transmission rates with USUV (80% and 69%, respectively) were significantly higher than with WNV (46% and 33%, respectively) under elevated temperature (28 °C) in these mosquitoes [101].

Two mosquito species of the genus *Aedes* were assessed for their vector competence to USUV, namely *Ae. Albopictus,* repeatedly found infected in northern Italy [106], and *Ae. japonicas*, which is invading Europe and disseminating USUV in Graz (Austria) [107]. North American and European populations of *Ae. albopictus* appeared to be experimentally incompetent vectors for USUV [102,106] and the detection of USUV from field-collected *Ae. albopictus* was explained by simple recent engorgement from viremic birds [102]. In contrast, field-collected *Ae. japonicus* mosquitoes from the Netherlands showed USUV-positive saliva after 14 days at 28 °C, and, therefore, could play a role in the transmission cycle of the virus in Europe [105].

A key step in flavivirus transmission and vector competence is crossing the midgut barrier, which acts as a physical and immune barrier that limits the replication and spread of the virus in the insect [108]. In this regard, the midgut acts as the major bottleneck for the dissemination of USUV, as female *Cx. pipiens* and *Ae. japonicas* intrathoracically injected with USUV showed higher transmission rates than those infected via the oral route [100,101,105]. The induction of antiviral responses, including small RNA pathways, is also a determinant of viral replication and dissemination after a blood meal of the female mosquito. USUV elicits a strong expression of RNA-derived small interfering RNAs (siRNAs), which are 21-nucleotide-sized RNA products from viral double-stranded RNA cleaved by the endoribonuclease Dicer-2 [101,105]. However, 25–30-nt Piwi-interacting RNAs (piRNAs) were not identified in USUV-infected *Ae. japonicas* [105] and *Cx. pipiens* [101], suggesting that siRNAs were the major group of small RNAs targeting USUV in these mosquitoes [101,105]. The induction of selective pressure may influence virus replication in mosquitoes, but there are currently no data concerning RNA hot spots during USUV infection in mosquitoes.

### 3.2. Bird Infection Models

USUV is highly pathogenic in some wild and captive bird species, due to its extensive tropism and virulence in various tissues and organs. Thus, these hosts are the most plausible in vivo models to characterize the pathogenesis of USUV infection. Besides, USUV has very selective pathogenicity within these hosts, including members from the same bird family. For instance, the natural USUV infection might be unapparent in domestic geese (*Anser anser f domestica*), while in another anatid, the common scoter (*Melanitta nigra*), USUV could result in fatal infection [7]. Thus, it would be tempting to use such models to identify molecular determinants associated with virulence and host tropism, which may help anticipate key events leading to the possible emergence of USUV in new hosts and territories. However, to date, only three avian species have been used to address the susceptibility of these hosts to USUV infection. Domestic chicken (*Gallus gallus domesticus*) and geese (*Anser anser f domestica)* were reported to resist USUV infection under experimental conditions [109]. More recently, the domestic canary (*Serinus canaria*), a passerine species, such as highly susceptible blackbirds, showed a mortality rate of 30% after infection via the intraperitoneal (IP) route with two different doses (10^3^ and 10^6^ TCID_50_) of a European strain of USUV [110]. In addition, USUV induced a specific humoral immune response in almost all the survivors after 15 days of infection [110]. Chicken and goose embryos were also tested for their susceptibility to the virus. While USUV showed viral replication in goose embryos tissues [111], some studies showed that chicken embryos were resistant to infection [5,73,79], while one recent paper demonstrated that they are highly susceptible to USUV infection in a dose-dependent manner [71]. These contradictory results could be explained by the genetic variability of the USUV strains and the differences in the genetic backbone of the eggs used, conditioning the immune response between breeds/individuals of the same bird species [71].

In addition to their susceptibility to USUV, the avian models available to date to study USUV, namely chicken and goose embryos and domestic canaries, have shed new light on USUV pathogenesis and transmission in birds. Similar to WNV, death due to USUV in domestic canaries was more likely attributed to a multi-systemic failure than to a pure neurologic disease, and the virus infected all major systems and a wide variety of cell types [110]. The myocardial cells strongly supported viral replication, as viral antigens were systematically detected by immunohistochemistry (IHC) in the experimentally infected chicken embryos and canaries [71,110].

In all these three models, USUV displayed a particular tropism for the eyes [71,110,111]. Visual impairment and ocular lesions have been described following infection of birds with other flaviviruses, such as WNV [112,113]. A vision assessment should be performed during future experimental infections in vivo with USUV.

Microscopically, despite the detection of USUV antigens by IHC in many embryonic tissues, USUV replication was not associated with any significant lesions in the goose embryos, while multifocal necrosis and non-suppurative inflammation were observed only in the Chorioallantoic membrane (CAM) of the chicken embryo [71]. In canaries, these lesions affected several organs, including the brain, which, however, showed no IHC labeling. Similarly, the relative amount of vRNA in the tissues of naturally infected birds collected in Belgium in 2017–2018 during passive surveillance of USUV circulation was not proportional to the intensity of the lesions [2]. Therefore, the importance of viral replication is not necessarily correlated with the intensity of the lesions in these hosts [2]. Alternatively, the pathological changes could, as in the case of WNV infection [114], be induced not only by direct viral replication but also, and maybe more significantly, by the exacerbated inflammatory response in the host. Further studies are required to consolidate these observations.

Excretion of USUV via the immature feathers of birds during the early stages of infection was demonstrated [110]. Similarly, during embryonic development in chicken, the virus replicated in feather follicles [71]. These preliminary observations suggest that feathers can potentially play a role in the spread of the virus. Furthermore, the excretion of relatively large vRNA amounts via the droppings during the five days following the canary infection was consistent with the detection of USUV antigens in the intestines of the naturally infected birds or experimentally infected chicken embryos and the kidneys of the naturally infected blackbirds [71,110]. The infectivity of the detected viral particles has, however, not been evaluated in cell culture. Nevertheless, the non-vector-borne transmission of WNV has been demonstrated experimentally via contaminated food, water, predation, or contact between infected and uninfected birds [115] and similar alternative routes for USUV transmission deserve to be investigated.

### 3.3. Mammalian Models

#### 3.3.1. Immunocompetent Models

Developing an animal model relevant to human USUV infection seems to be extremely challenging because experimental infections have shown that immunocompetent mammals rarely develop severe forms of USUV disease. African fruit bats (*Eidolon helvum*) and (*Rousettus aegyptiacus*) and the Angolan free-tailed bat (*Tadarida (Mops) condylura*) were not susceptible at all to USUV injected intraperitoneally [116]. Guinea pigs showed only an antibody response following intracerebral inoculation with the USUV SAAR-1776 strain [117]. The Abyssinian grass rat (*Arvicanthis abyssinicus*) could exhibit a trace of viremia 1–2 days after IP inoculation of USUV (unknown strain) and developed neutralizing antibodies [117]. Immunocompetent mouse models showed different susceptibilities to USUV infection across the studies (Table 5).

Intracerebral (IC) inoculation of USUV successfully induced signs and mortalities in neonatal and 3–4 weeks-old immunocompetent mice [18,46]. However, this injection route is not pertinent enough to describe USUV neuropathogenicity, as it only models viral neurovirulence. Thus, peripheral inoculation (e.g., subcutaneous SC or IP) was more commonly used to reflect both USUV neurovirulence and neuroinvasiveness [123]. Experimentally, no mortality was observed following IP infection with USUV of Naval Medical Research Institute (NMRI) mice aged over 2 weeks with a European USUV strain [122]. Similarly, the USUV prototype strain SAAR-1776 showed no pathogenicity in adult Swiss mice via the IP route [18,120,121]. However, in the study of Diagne et al. [46], both SC and IP infections of this strain resulted in a 30% and 50% mortality, respectively, in 3–4-week-old Swiss Webster (CFW) mice after 15 days of infection [46]. Likewise, in the same study, the IP inoculation of a mouse-derived USUV strain induced a 10% mortality 10 days after infection [46]. USUV infection failed to elicit pathogenicity in wild-type 129/Sv mice via the IP [118,119] and IN routes [118] but induced a typical neurological disease in a single 129/Sv mouse infected via the ID route [118]. These findings indicate that the outcome of USUV infection in immunocompetent mice depends on several factors, such as the strain of virus or mouse used. Age is also a key determinant of susceptibility to USUV and suckling mice are generally much more susceptible than older animals. NMRI suckling mice showed 100% mortality with as few as 10^3^ Plaque-forming units (PFU) after 11 days of infection [122]. Dose-dependent mortality was observed in Swiss suckling mice, as 84% and 40% survived the infection with 10^2^ and 10^4^ PFU, respectively [85,120]. The higher predisposition of newborn neurons to apoptosis and the incomplete development of the BBB are plausible explanations for this difference in the infection outcome [122].

Although immunocompetent models present limitations regarding their efficiency to manifest the USUV-associated disease, they are important to obtain knowledge about USUV pathogenesis under functional innate and adaptive immune responses of the host.

In immunocompetent mice, USUV infection induced clinical signs, such as disorientation, depression, paraplegia, and paralysis, associated with extensive neuronal death, including both necrosis and apoptosis in the brain [118,122]. Alternatively, no trace of viral infection [120] or a simple detection of the USUV genome in brain portions of USUV-infected mice were described after 15 days post-infection, without the induction of specific clinical signs [118]. These models reflect the infection in humans, in which most individuals show subclinical infections but rare cases can develop clinical disease.

While the skeletal muscle has been identified as the only site of peripheral viral replication in NMRI suckling mice [122], USUV infected and replicated in various tissues and organs, including the eyes of Swiss suckling mice [85]. These studies also showed that USUV targets the spinal cord. Infection of white matter cells with resulting apoptosis and demyelination were detected in the NMRI newborn mice and viral replication was suggested to trigger myelin breakdown and oligodendrocyte damage [122]. An inflammatory response in the spinal cord with the presence of similar cytokines released in the brain was described in Swiss neonatal mice [85]. RNA shedding via the urine in the latter model appeared 6 days after the infection and persisted beyond day 12 post-infection [85]. These viral particles were not assessed for their infectivity in cell culture and further investigations are needed, as for birds, to investigate a potential indirect transmission of USUV.

The study of the antibody response to USUV showed the induction of detectable IgG against the virus by 15 days post-infection [118,120,121]. The neutralizing capacity of these specific antibodies was assessed in one study and none of the USUV-IgG-positive samples efficiently neutralized USUV [120]. Cross-protective antibodies induced by WNV or USUV infections were also investigated. Previous USUV infection has been shown to reduce mortality in adult female Swiss mice or suckling mice following the challenge with a virulent strain of WNV [120]. In vitro, while all sera from WNV-infected animals cross-reacted with USUV by day 17 post-infection, no cross-reactivity against WNV was observed with sera from USUV-infected Swiss adult mice at any time post-infection [120]. This finding confirms the different antibody responses elicited against the two viruses, underlying the higher susceptibility of this model to WNV [120]. Levels of IgG anti-USUV in Swiss mice were greater after immunization with a recombinant WNV-subviral particles-based vaccine (WNV-RSPs) than with USUV itself. Protection studies of mice immunized with WNV-RSPs against USUV challenge were, however, hampered due to the absence of an immunocompetent lethal challenge model for USUV [121].

#### 3.3.2. Immune-Deficient Models

Contrary to immunocompetent mice, immune-deficient mice display signs of USUV disease and high mortality rates following their infection with USUV (Table 6). The first model used AG129 mice, which are deficient in both IFN-α/β and IFN-γ receptors, making them unresponsive to type I and II IFNs. A dose-dependent survival rate was observed in this very susceptible USUV model, as 10 PFUs were able to induce a 67% mortality rate after one week [79]. Prior to death, symptoms including rapid bodyweight loss, conjunctivitis, and lower limb paralysis were observed [79]. USUV RNA was found in the blood, brain, and other organs (spleen, kidney, liver, intestine), denoting widespread viral replication. Treatment of mice with favipiravir significantly reduced the viral load in blood and tissues and significantly delayed virus-induced disease [79].

More recently, mice with an impaired type 1 IFN signaling (*Ifnar1^−/−^*) on a C57BL/6 background were used to study USUV neuropathogenesis [85]. A massive inflammatory response in the brain and spinal cord was recorded, including the secretion of the chemokine CXCL10, which attracts leukocytes into the CNS and is suggested to play a pivotal role in USUV neuropathogenesis. Along with USUV replication, a strong innate antiviral response induction was detected, in agreement with previous in vitro studies suggesting that USUV does not interfere efficiently with the IFN response pathway [82,85,86,87]. Detection of USUV-RNA in the eyes of mice and histopathological changes in the retina, such as disruption of both inner and outer structures and the loss of photoreceptors [85], are in accordance with previously described USUV ocular tropism in birds [71,110,111].

## 4. Conclusions and Perspectives

Recently, a succession of arboviral epidemics and epizootics around the world has drawn the attention of the scientific community to the significant threat posed by these emerging pathogens to public and animal health. Among these arboviruses, USUV is a neurotropic flavivirus primarily circulating in Africa, which has spread to a large part of the European continent. USUV is genetically close to major mosquito-borne flaviviruses for humans, such as WNV, Japanese encephalitis virus, Dengue virus, and ZIKV. It can be currently considered as a leading model for the study of flaviviral pathogenesis and the development of prophylactic and therapeutic solutions against these more pathogenic flaviviruses. Indeed, it can be handled under level 2 biosafety conditions; besides, field strains are easily accessible and have a certain degree of natural genetic variation. Despite these advantages, little effort has been made so far to the development of in vitro and in vivo models for the study of this neurotropic virus, given that human infections most often remain asymptomatic, or with a benign clinical expression and only a few bird species naturally develop severe forms of USUV virus disease. However, because the incidence of USUV infection in humans is on the rise [97,124], the virus could spread to the American continent [102], and USUV strains with enhanced neurovirulence could potentially emerge, scientists should anticipate a USUV epidemic in humans in the future and, thus, consider continuous monitoring of USUV circulation and validation of USUV research models. USUV experiments in birds are irreplaceable to investigate the specific pathogenicity of field strains, virus transmission routes, and host tropism. In the same context, more mosquito species and populations should be investigated for their vector competence to USUV. Further studies are warranted to assess susceptibility to USUV infection in non-human primates, as recent evidence showed that they could also become infected by the virus [55] and, thus, could be a very relevant USUV infection model for human infection. Besides, the validation of novel cell culture models, such as 3-D models, and the generation of recombinant USUV using reverse genetics can be instrumental to further characterize the infection and to develop more effective vaccines and antiviral therapies.

## Figures and Tables

**Table 1:** List of animal cells used for the characterization of Usutu virus (USUV).

Species		Cells		USUV Strain (GenBank)	Infection	Cytopathic Effects	Refs
**Armadillo** *Dasypus novemcinctus*		Trachea fibroblasts DNl.Tr, ATCC: CRL-6009		AY453412	+	-	[77]
**Bat** *Tadarida brasiliensis*		Lung epithelial cells Tb 1 Lu *, ATCC: CCL-88		AY453412	+	-	[77]
**Birds**	Chicken *Gallus gallus domesticus*	Chicken embryo fibroblasts	Primary cell culture	AY453411	-	-	[73]
			DF-1, ATCC: CRL-12203	AY453412	+	-	[77]
		Chorioallantoic membrane primary cells		AY453411, MK230892 MK230891, KX555624	+	+	[71]
	Goose *Anser anser domesticus*	Primary culture of embryo fibroblasts GEF		AY453411	+	+	[73]
**Cat** *Felis catus*		Kidney epithelial cell line CRFK	ATCC: CCL-94	AY453412	+	+	[77]
			-	AY453411	+	+	[73]
**Cow** *Bos taurus*		Turbinate cells BT, ATCC: CRL-1390		AY453412	+	-	[77]
		Madin-Darby bovine kidney cell line MDBK, ATCC: CCL-22		AY453411	+	-	[73]
**Deer** *Odocoileus hemionus*		Kidney fibroblasts OHH1.K, ATCC: CRL-6193		AY453412	+	+	[77]
**Dog** *Canis familiaris*		Madin-Darby kidney epithelial cells MDCK	ATCC: CCL-34	AY453412	+	-	[77]
			-	AY453411	+	-	[73]
		Kidney epithelial cell line DK*, ATCC: CRL-6247		AY453411	+	-	[73]
**Fox** *Urocyon cineroargenteus*		Lung fibroblasts FoLu, ATCC: CCL-168		AY453412	+	+	[77]
**Hamster** *Mesocricetus auratus*		Baby hamster kidney fibroblasts BHK-21 (ATCC: CCL-10) and BF cell lines		AY453411	+	-	[73]
**Horse** *Equus caballus*		Dermal fibroblasts cell line	ED	AY453411	+	-	[73]
			E.Derm ATCC: CCL-57	AY453412	+	+	[77]
		Primary horse kidney cells EqK		AY453411	+	-	[73]
**Mink** *Neovison vison*		Lung epithelial cells Mv 1 Lu, ATCC: CCL-64		AY453412	+	+	[77]
**Monkey**	*Cercopithecus aethiops*	Kidney epithelial cell line Vero **	-	AY453412, AY453411	+	+	[75]
			-	LT854220	+	+	[11]
			-	AY453411	+	+	[78]
			Persistently infected	AY453411	+	-	[76]
			Vero E6, ATCC: CRL-1586	KJ438730	+	+	[79]
			-	AY453412	+	+	[80]
			B4	0679/2006	+	+	[81]
	*Macaca mulatta*	Kidney epithelial cells LLC-MK2, ATCC: CCL-7		AY453412	+	+	[77]
**Mouse** *Mus musculus*		Primary cultures of astrocytes, microglial cells, and neurons		AY453411	+	+	[82]
**Mosquito** *Aedes albopictus*		Larvae C6/36 cell line ATCC: CRL-1660		JF330418 (Germany, *Cx. p*.) AY453412, KC754955 MH727238, MH727239 MH727241, MH727240 MH727242	+	+	[33,46]
**Opossum** *Didelphis virginiana*		Kidney epithelial cells OK, ATCC: CRL-1840		AY453412	+	+	[77]
**Pig** *Sus scrofa*		Primary monocyte-derived dendritic cells MoDC *		AY453412	+	+	[83]
		Epithelial kidney cell line PK-15, ATCC: CCL-33		AY453412 AY453411	+	+	[72,73,77]
**Rabbit**	*Oryctolagus cuniculus*	Kidney epithelial cell line RK-13, ATCC: CCL-37		AY453411	+	-	[73]
	*Sylvilagus floridanus*	Skin epidermis cells Sf 1 Ep, ATCC: CCL-68		AY453412	+	+	[77]
**Raccoon** *Procyon lotor*		Uterus fibroblasts Pl 1 Ut, ATCC: CCL-74		AY453412	+	-	[77]
**Rat** *Rattus norvegicus*		Brain glial tumor cell line C6, ATCC: CCL-107		AY453411	+	-	[73]
**Turtle** *Terrapene carolina*		Epithelial heart cell line TH-1, ATCC: CCL-50		AY453411	+	-	[73]
**Vole**	*Myodes glareolus*	Epithelial kidney cell line BVK168, CCLV-RIE 1313		0679/2006	+	+	[81]
				HE599647	+	+	[84]
		Lung cell line MGLU-2-R, CCLV-RIE 1304		HE599647	-	-	
	*Microtus arvalis*	Kidney cell line FMN-R, CCLV-RIE. 1102			+	-	
		Brain cell line FMG-R, CCLV-RIE 1129			-	-	
**Woodchuck** *Marmota monax*		Liver epithelial cell line WCH-17 *, ATCC: CRL-2082		AY453412	+	-	[77]

* Very low replication of USUV. ** Most commonly used in virus replication for direct diagnosis (viral isolation) and amplification or serological assays (seroneutralization). Abbreviation: *Cx*. *p: Culex pipiens.*

**Table 2:** List of human cell lines used for the characterization of Usutu virus (USUV).

Cell Lines		USUV Strain (GenBank)	Infection	Cytopathic Effects	Other Findings	Ref
Primary astrocytes		AY453411	+	-	USUV decreases cellular proliferation without induction of apoptosis, targets human astrocytes and upregulates antiviral genes more efficiently than ZIKV	[82]
		LT854220	+	-	-	[11]
Brain-like primary endothelial cells (hBLECs)		KX601692	+	-	Secretion of chemokines such as CXCL10 or CCL5	[85]
Colon adenocarcinoma cell line CaCo-2, ATCC HTB-37		AY453411	+	-	-	[86]
Colon adenocarcinoma grade cell line II HT29, ATCC HTB-38						
Colon adenocarcinoma cell line SW480, ATCC CCL-228						
Embryonic lung cell line MRC-5, ATCC CCL-171						
Epidermoid larynx carcinoma cell line Hep-2, ATCC CCL-23		AY453411	+	+	An established USUV infection can overcome the antiviral effect of types I and III IFNs	[86]
Epidermoid oral carcinoma cell line KB, ATCC CCL-17			+	+	-	
Epitheloid cervix carcinoma HeLa, ATCC CCL-2		AY453411	+	-	-	[73]
		AY453411	+	-	-	[86]
Hepatoblastoma cell line Hep-G2, ATCC HB-8065		AY453411	+	-	-	[86]
Lung adenocarcinoma epithelial cell line A549, ATCC CCL-185		AY453411	+	n.i.	USUV replication was lower than that of WNV in the presence of a large variety of subtypes of IFN-α, -β and γ	[87]
		AY453411	+	+	-	[86]
Primary monocyte-derived dendritic cells (DC)	hMoDC	AY453412	+	+	High IFN-β and TNF responses were found	[83]
	(DCs)	AY453411	+	n.i.	USUV induced a higher activation of IFN-associated response and was more sensitive to types I and III IFN than WNV	[87]
Induced pluripotent stem cells (iPSc)-derived neural stem cells (NSCs)		AY453411	+	+	Cells undergo cellular death by caspase 3-dependant apoptosis	[82]
		AY453411	+	+*	USUV replicated less efficiently and induced less inflammatory response and cell damage than WNV	[88]
Induced pluripotent stem cell (iPSC)-derived retinal pigment epithelium RPE		KX601692	+	-	Strong antiviral and pro-inflammatory response	[85]
Primary nasal epithelial cells (NECs)		AY453412	+	-	USUV did not induce a significant IFN response and inflammatory mediators IL-8/CXCL8 and IP-10/CXCL10 release in comparison to mock controls	[89]
Vascular endothelial cells EA.hy.926, ATCC: CRL-2922		AY453412	+	+	-	[77]

* Mild cytopathic effects. Abbreviations: IL: Interleukin; IFN: Interferon; n.i.: Not indicated; TNF: Tumor necrosis factor; WNV: West Nile virus.

**Table 3:** Infection studies with USUV in European mosquito models.

Study	Mosquito Species	Virus Strains	Infective Dose(s) and Route	Incubation Temperature	Infection Rate	Transmission Rate	Dissemination Rate	Conclusions
[100]	*Cx. p. molestus* *Cx. p. pipiens* (The Netherlands)	MH891847	10^7^ TCID_50_/mL Oral route	28 °C	Day 14 p.i: 66.6% (34/51) and 87.9% (58/66) respectively	Day 14 p.i: 31.4% (16/51) and 21.2% (14/66) respectively	-	The tested species/ biotypes are competent vectors for USUV and the midgut barrier restricts virus dissemination in the mosquito after oral exposure
			3.5 × 10^3^ TCID_50_/mL Intrathoracic route		Day 14 p.i: 100% (18/18) and (17/17) respectively	Day 14 p.i: 94% (17/18) and 88% (15/17) respectively	-	
[105]	*Ae. japonicus* (Lelystad, the Netherlands)	MH891847	1.6 × 10^7^ TCID_50_/mL Oral route	28 °C	Day 14 p.i: 13.3% (4/30)			
			3.5 × 10^3^ TCID_50_/mL Intrathoracic route		Day 14 p.i: 100% (26/26)	Day 14 p.i: 88.5% (23/26)	Day 14 p.i: 100% (26/26)	
[103]	*Cx. p.* typical form (Caldbeck: UK)	AY453412	10^6^ PFU/mL Oral route	25 °C	Days 7 p.i: 0/20 (0%) Day 14 p.i: 1/20 (5%) Day 21 p.i: 1/7	Day 7 p.i: 0/20 (0%) Day 14 p.i: 1/1 Day 21 p.i: 0/7	Day 7 p.i: 3/20 (15%) Day 14 p.i: 1/20 (5%) Day 21 p.i: 0/7	Limited susceptibility to infection with USUV
	*Cx. p.* hybrid form (Brookwood, UK)				Days 7 and 14 p.i: (0%) Day 21 p/i: 1/18 (5.5%)	Days 7 and 14 p.i: (0%) Day 21 p/i: 0/18 (0%)	Days 7 and 14 p.i: (0%) Day 21 p/i: 0/18 (0%)	
[106]	*Ae. albopictus* (Emilia-Romagna, Italy)	KF055442 (E2, Italy, *T. m*.)	0.66 × 10^7.5^ TCID_50_ Oral route	28 ± 1 °C	0% after 96 h, 1 and 2 weeks	0% after 96 h, 1 and 2 weeks of infection		*Ae. albopictus* has a low vector competence for USUV
		KF055441 (E2, Italy, *Cx*. *p.*)			0% after 96 h, and 2 weeks Day 7p.i: 1/6			
		KF055440 (E2, Italy, *Cx*. *p.*)	0.66 × 10^7.9^ TCID_50_ Oral route	28 ± 1 °C	0% after 96 h, 1 and 2 weeks			
[101]	*Cx. p.* (Brummen, The Netherlands)	HM569263	4 × 10^7^ TCID_50_/mL Oral route	28 °C	80%	69%	-	Temperature affects the susceptibility of *Cx. pipiens* to USUV infection and the midgut barrier restricts virus dissemination in the mosquito after oral exposure
			5.5 × 10^3^ TCID_50_/mL Intrathoracic route		100%	100%		
			3.2 × 10^7^ TCID_50_ /mLOral route	18 °C	11%	-		
				23 °C	53%			
				28 °C	90%			

Abbreviations: *Cx*. *p.: Culex pipiens;* E2: Europe 2; PFU: Plaque-forming unit; p.i.: Post-infection; TCID_50_: 50% Tissue infective dose.

**Table 4:** Infection studies with USUV in non-European mosquito models.

Study *	Mosquito Species		Infective Dose(s)	Incubation Temperature	Infection Rate	Transmission Rate	Dissemination Rate	Conclusions
[102]	*Cx. pipiens*	American population (Mercer County, NJ, USA)	10^7.52^ TCID_50_/mL	28 °C	Day 7 p.i: 25% (4/16) Day 14 p.i: 58.6% (17/29)	Day 14 p.i: 23.5% (4/17)	Day 7 p.i: (1/1) Day 14 p.i: 93.3% (12/13)	*Cx. pipiens* complex mosquitoes are susceptible to USUV and competent for its potential transmission in North America *Ae. albopictus* is highly refractory to USUV infection and unlikely to contribute to USUV transmission in North America
	*Ae. albopictus*				0% at days 7 and 14 p.i	-	-	
	*Cx. quinquefasciatus* American population (Vero Beach, FL, USA)		10^6.95^ TCID_50_/mL		Day 7 p.i: 93.3% (14/15) Day 14 p.i: 70% (21/30)	Day 14 p.i: 19% (4/21)	Day 7 p.i: (4/6)4 Day 14 p.i: 35.7% (5/14)	
[104]	*Cx. neavei* African population (Barkedji, Senegal)		2 × 10^7^ PFU/mL (triplicate)	27 °C	Day 14 p.i: 1/3, 2/9 and 0/1	-	Day 14 p.i: 0/3	Dose-dependent vector competence in *Cx. neavei*
			2 × 10^8^ PFU/mL		Day 14 p.i: p40/44 (90.9%)	Day 14 p.i: 13/16 (81.3%)	Day 14 p.i: 16/40 (40.0%)	
[96]	*Cx. neavei* African population		10^6^ PFU/mL	26 °C	Day 14 p.i: 2/10 (20%)	-	-	Failure of virus transmission to hamsters

* USUV strain: AY453412 (A2, South Africa, *Cx. neavei*) ingested in blood meals.

**Table 5:** Immunocompetent mouse models for studying USUV biology.

Study	Strains	Sex	Age (Weeks)	Viral Strains (GenBank)	Doses and Routes of Inoculation	Results
[85]	Swiss	M	1	KX601692	10^4^ TCID_50_ IP	Weight loss/failure to gain weight/limb weakness and hind-limb paralysis/mortality of 60% at 14 days post-infection. Massive inflammatory response in the CNS and eyes (neuroretinitis and uveitis)/viral genome identification by RT-qPCR at 6 dpi in the liver, spleen, hind limb muscle, kidney, bladder and especially in nervous tissues such as eyes (including optic nerve), brain, spinal cord, and sciatic nerves
[118]	129/Sv	F	4–5	MK230891 MK230892	10^6^ TCID_50_ IP, ID or IN	Disorientation/paraplegia/neuronal death in the brain and spinal cord and systemic RNA detection in a single mouse. Viral RNA detected in the brain 15 days post-infection of mice after IN injection of both strains/variable antibody-response.
[46]	Swiss Webster (CFW)	NI	3–4	KC754955 AY453412 MH727238	10^3^ PFU IC	Weight loss/tremors, apathy and paralysis of the posterior limbs 4 days after infection 100% mortality between the 8th and the 10th day
					10^3^ PFU IP	Mortality of 1/10 at 10 days post-infection with KC754955. 60% of morbidity and 50% of mortality at 15 days post-infection with AY453412
					10^3^ PFU SC	No effect after the injection of MH727238. No effect after the inoculation of KC754955 or MH727238. Weight loss and 30% mortality at 15 days post-infection with AY453412.
[119]	129/Sv	M/F	6	KU760915 *	10^4^ PFU IP	No signs nor mortalities.
[120]	Swiss	F	8	AY453412	10^2^ ou 10^4^ PFU IP	No signs/mortalities/neutralizing antibodies/USUV-RNA in the organs (e.g., brain) at any tested time after infection (4 to 35 days).
			1			Dose-dependent mortality (15.8% and 60% respectively). USUV-RNA detection in the brain/anti-USUV IgG antibodies were detected from 15 d.p.i.
[121]	Swiss	F	10	AY453412	10^4^PFU IP	No signs nor mortalities.
[122]	NMRI	NI	1	AY453411	10^3^ TCID_50_ IP	Disorientation, paraplegia, paralysis 100% of mortality after 11 days of infection. Neuronal and glial cells apoptosis/neuronal demyelination.
			>1			No signs nor mortalities.
[18]	Swiss	NI	Suckling mice	ENT MP 1626	IC (isolation and reisolation of the viral strain from *Mansonia* (*Coquillettidia*) *aurites* mosquitoes in Uganda)	Clinical signs (details not given, except paralysis in one mouse) and death by day 14 in 6/9 newborn mice.
			5–6		>10^6,5^ TCID_50_ IP	No mortalities in adult mice.

* Derivative of the strain AY453412 by passages on cells. Abbreviations: dpi: days post-infection; NI: Not indicated; M: Male; F: Female; IC: Intra-cerebral; ID: Intra-dermal; IN: Intra-nasal; IP: Intra-peritoneal; NMRI: Naval Medical Research Institute; SC: Subcutaneous; TCID_50_: 50% Tissue culture infective dose; WNV-RSPs: West Nile virus-Recombinant Sub-viral particles.

**Table 6:** Immunocompromised mouse models for studying USUV biology.

Study	Strains	Sex	Age (Weeks)	Viral Strains (GenBank)	Doses and Routes of Inoculation	Results
[85]	C57BL/6 ^1^	M	8–12	KX601692	10^4^ TCID_50_ IP	Weight loss/failure to gain weight/ limb weakness and hind-limb paralysis/massive inflammatory response in the CNS and eyes (neuroretinitis and uveitis); All infected C57BL/6 mice died 6 days post-infection
[79]	AG129 ^2^	NI	8–14	KJ438730	10^1^, 10^2^, 10^3^, 10^4^, 10^5^, 10^6^ PFU IP	75–100% mortality/Weight loss, apathy, conjunctivitis, and neurological symptoms (mobility disorders, paralysis of the lower limbs). Treatment of mice with favipiravir (150 mg/kg/dose, oral route) significantly reduced viral load in blood and tissues and significantly delayed virus-induced disease.
[119]	129 SvEv ^1^	M and F	6	KU760915 ^3^	10^4^ PFU IP	Ruffled fur, hunching and ataxia/89% mortality at day 10 post-infection. Genomic USUV RNA detection in brain samples from dead animals. Significantly higher survival rate after USUV challenge in mice inoculated with pcDNA-USUV ^4^ (66.7%) than those inoculated with the empty vector (18.2%). The pcDNA-USUV can prime USUV specific humoral response.

^1^ Type 1 IFN-deficient mice; ^2^ Type 1 and 2 INF-deficient mice; ^3^ USUV-BIOTEC: Derivative of USUV prototype strain SAAR-1776 (GenBank: AY453412) by passages on cells; ^4^ Plasmid pcDNA-USUV encoding the complete sequence of USUV-BIOTEC membrane precursor prM and envelope E proteins; Abbreviations: M: Male; F: Female; IP: Intra-peritoneal; TCID_50_: 50% Tissue culture infective dose; NI: Not indicated.
